# 1,3,5,7-Tetra­bromo­adamantane

**DOI:** 10.1107/S1600536810054474

**Published:** 2011-01-08

**Authors:** You-Ming Zhang, Cheng Cao, Yan-Yun Lu, Qin-Sheng Zhang, Tai-Bao Wei

**Affiliations:** a Department of Chemistry, Provincial Key Laboratory of Characteristic Resources Utilization of Gansu Corridor, Hexi University, Zhangye 734000, People’s Republic of China; b College of Chemistry and Chemical Engineering, Key Laboratory of Eco-Environment-Related Polymer Materials of the Ministry of Education, Gansu Key Laboratory of Polymer Materials, Northwest Normal University, Lanzhou 730070, People’s Republic of China

## Abstract

In the pyramidal-shaped mol­ecule of the title compound, C_10_H_12_Br_4_, the four terminal Br—C bond distances are nearly identical, ranging from 1.964 (4) to 1.974 (4) Å. The Br⋯Br distance of 3.6553 (7) Å indicates van der Waals contacts between mol­ecules in the crystal structure.

## Related literature

For applications of adamantane compounds, see: Kim *et al.* (2001 ▶); Kozhushkov *et al.* (2005 ▶); Li *et al.* (2003 ▶). For related structures, see: Pedireddi *et al.* (1994 ▶); Reddy *et al.* (1995 ▶). For the synthesis, see: Murray *et al.* (1989 ▶); Migulin & Menger (2001 ▶).
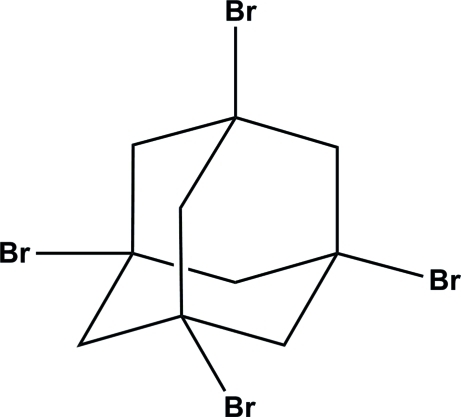


## Experimental

### 

#### Crystal data

C_10_H_12_Br_4_
*M* *_r_* = 451.84
Monoclinic, 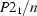

*a* = 11.7669 (4) Å
*b* = 9.0612 (3) Å
*c* = 12.1493 (4) Å
β = 98.529 (2)°
*V* = 1281.06 (7) Å^3^
*Z* = 4
Mo *K*α radiation
μ = 12.53 mm^−1^
*T* = 296 K
0.35 × 0.32 × 0.24 mm

#### Data collection

Bruker APEXII CCD diffractometer
Absorption correction: multi-scan (*SADABS*; Sheldrick, 2000 ▶) *T*_min_ = 0.097, *T*_max_ = 0.153
7087 measured reflections
2511 independent reflections
1892 reflections with *I* > 2σ(*I*)
*R*_int_ = 0.047

#### Refinement

*R*[*F* ^2^ > 2σ(*F* ^2^)] = 0.033
*wR*(*F* ^2^) = 0.077
*S* = 1.01
2511 reflections
127 parameters
H-atom parameters constrained
Δρ_max_ = 0.70 e Å^−3^
Δρ_min_ = −0.67 e Å^−3^

### 

Data collection: *APEX2* (Bruker, 2007 ▶); cell refinement: *SAINT* (Bruker, 2007 ▶); data reduction: *SAINT*; program(s) used to solve structure: *SHELXTL* (Sheldrick, 2008 ▶); program(s) used to refine structure: *SHELXTL*; molecular graphics: *SHELXTL*; software used to prepare material for publication: *SHELXTL*.

## Supplementary Material

Crystal structure: contains datablocks I, global. DOI: 10.1107/S1600536810054474/xu5115sup1.cif

Structure factors: contains datablocks I. DOI: 10.1107/S1600536810054474/xu5115Isup2.hkl

Additional supplementary materials:  crystallographic information; 3D view; checkCIF report

## Figures and Tables

**Fig. 1.**
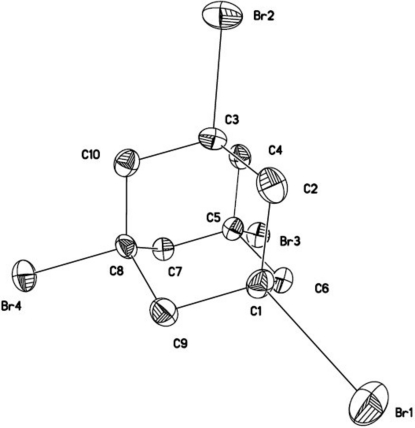
The structure of the title compound, showing the atom-labelling scheme. Displacement ellipsoids are drawn at the 30% probability level. H atoms have been omitted for clarity.

**Fig. 2.**
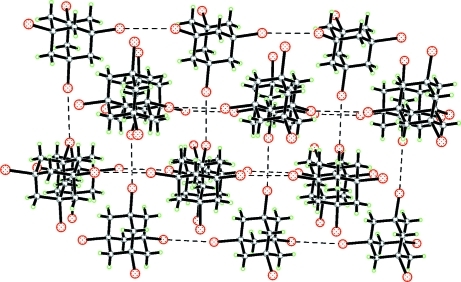
The three-dimensional packing diagram of the compound by intermolecular Br···Br interactions.

## supplementary crystallographic information

### Comment

Derivatives of adamantane attract a broad interdisciplinary interest as rigid molecular scaffolds for sustaining the structures of polyfunctional species, which find various applications in the chemistry of supramolecular systems, macromolecules, dendrimers and polymers (Kim *et al.*, 2001; Kozhushkov *et al.*, 2005; Li *et al.*, 2003). Thus, adamantanes substituted in the four available bridgehead positions represent a family of rigid tetrahedral building blocks for the synthesis of hydrogen and coordination-bonded framework polymers, and they are paradigmatic for the general principles of crystal design.

The asymmetric unit contain only a 1,3,5,7-Tetrabromoadamantane molecule. The molecular structure is shown in Fig. 1. The conformation of the 1,3,5,7-Tetrabromoadamantane unit is very similar to the conformation in the crystal structure of adamantane and 1,3,5,7–tetraiodoadamantane (Pedireddi *et al.*, 1994; Reddy *et al.*, 1995), with four nearly identical C–Br bons distance [1.967 (4), 1.969 (4), 1.974 (4), 1.964 (4) Å].

In the crystal structure, the intermolecular Br···Br distance is 3.655, 3.724, 3.884, 3.962 Å, respectively. Each molecule is joined to two or three others with Br···Br interactions leading to the crystal packing in a supramolecular 3-dimentional network as shown in Fig. 2.

### Experimental

The compound was prepared in the procudure reported by Murray *et al.* (1989) and by Migulin & Menger (2001). Adamantane (27.0 g, 0.2 mol) was added portionwise over 30 min to a stirred mixture of bromine (350 g, 2.2 mol) and anhydrous aluminium chloride (27.0 g, 0.2 mol) at 278 to 283 K. The mixture was heated to 363 K over a period of 1 h and held at that temperature for 24 h. Hydrogen bromide was evolved copiously during the addition and heating. Excess bromine (180 g) was distilled on the water bath. The residue was triturated with aqueous sodium sulfite (to remove excess bromine) with hydrochloric acid added (to dissolve aluminium salts). The solids were removed by filtration, washed, air-dried, and recrystallization from 1200 ml of glacial acetic acid.

### Refinement

The H atoms were placed at calculated positions with C—H = 0.97 Å and refined in riding model approximation with *U*_iso_(H) = 1.2*U*_eq_(C).

### Crystal data

**Table:**

C_10_H_12_Br_4_	*F*(000) = 848
*M**_r_* = 451.84	*D*_x_ = 2.343 Mg m^−^^3^
Monoclinic, *P*2_1_/*n*	Mo *K*α radiation, λ = 0.71073 Å
Hall symbol: -P 2yn	Cell parameters from 2005 reflections
*a* = 11.7669 (4) Å	θ = 2.6–25.9°
*b* = 9.0612 (3) Å	µ = 12.53 mm^−^^1^
*c* = 12.1493 (4) Å	*T* = 296 K
β = 98.529 (2)°	Block, colourless
*V* = 1281.06 (7) Å^3^	0.35 × 0.32 × 0.24 mm
*Z* = 4	

### Data collection

**Table:**

Bruker APEXII CCD diffractometer	2511 independent reflections
Radiation source: fine-focus sealed tube	1892 reflections with *I* > 2σ(*I*)
graphite	*R*_int_ = 0.047
φ and ω scans	θ_max_ = 26.0°, θ_min_ = 2.3°
Absorption correction: multi-scan (*SADABS*; Sheldrick, 2000)	*h* = −13→14
*T*_min_ = 0.097, *T*_max_ = 0.153	*k* = −11→9
7087 measured reflections	*l* = −14→14

### Refinement

**Table:**

Refinement on *F*^2^	Primary atom site location: structure-invariant direct methods
Least-squares matrix: full	Secondary atom site location: difference Fourier map
*R*[*F*^2^ > 2σ(*F*^2^)] = 0.033	Hydrogen site location: inferred from neighbouring sites
*wR*(*F*^2^) = 0.077	H-atom parameters constrained
*S* = 1.01	*w* = 1/[σ^2^(*F*_o_^2^) + (0.0333*P*)^2^ + 0.419*P*] where *P* = (*F*_o_^2^ + 2*F*_c_^2^)/3
2511 reflections	(Δ/σ)_max_ = 0.001
127 parameters	Δρ_max_ = 0.70 e Å^−^^3^
0 restraints	Δρ_min_ = −0.67 e Å^−^^3^

### Special details

**Table:**

Geometry. All e.s.d.'s (except the e.s.d. in the dihedral angle between two l.s. planes) are estimated using the full covariance matrix. The cell e.s.d.'s are taken into account individually in the estimation of e.s.d.'s in distances, angles and torsion angles; correlations between e.s.d.'s in cell parameters are only used when they are defined by crystal symmetry. An approximate (isotropic) treatment of cell e.s.d.'s is used for estimating e.s.d.'s involving l.s. planes.
Refinement. Refinement of *F*^2^ against ALL reflections. The weighted *R*-factor *wR* and goodness of fit *S* are based on *F*^2^, conventional *R*-factors *R* are based on *F*, with *F* set to zero for negative *F*^2^. The threshold expression of *F*^2^ > σ(*F*^2^) is used only for calculating *R*-factors(gt) *etc*. and is not relevant to the choice of reflections for refinement. *R*-factors based on *F*^2^ are statistically about twice as large as those based on *F*, and *R*- factors based on ALL data will be even larger.

### Fractional atomic coordinates and isotropic or equivalent isotropic displacement parameters (Å^2^)

**Table:**

	*x*	*y*	*z*	*U*_iso_*/*U*_eq_	
Br1	1.09687 (5)	0.62981 (5)	0.32537 (5)	0.05014 (17)	
Br2	1.16582 (4)	0.01485 (5)	0.39605 (5)	0.05204 (18)	
Br3	0.71851 (4)	0.26097 (6)	0.35978 (5)	0.05166 (17)	
Br4	0.94466 (5)	0.22452 (6)	−0.02140 (4)	0.04818 (16)	
C1	1.0321 (4)	0.4324 (5)	0.2896 (4)	0.0313 (10)	
C2	1.1152 (4)	0.3177 (5)	0.3482 (4)	0.0366 (11)	
H2A	1.1892	0.3251	0.3226	0.044*	
H2B	1.1262	0.3336	0.4280	0.044*	
C3	1.0625 (3)	0.1664 (4)	0.3203 (4)	0.0311 (10)	
C4	0.9471 (4)	0.1532 (5)	0.3643 (4)	0.0365 (11)	
H4A	0.9144	0.0558	0.3487	0.044*	
H4B	0.9576	0.1691	0.4441	0.044*	
C5	0.8681 (4)	0.2707 (5)	0.3050 (4)	0.0335 (10)	
C6	0.9174 (4)	0.4244 (5)	0.3321 (4)	0.0352 (11)	
H6A	0.8656	0.4991	0.2963	0.042*	
H6B	0.9279	0.4411	0.4118	0.042*	
C7	0.8488 (3)	0.2456 (5)	0.1789 (4)	0.0341 (11)	
H7A	0.8154	0.1490	0.1614	0.041*	
H7B	0.7971	0.3198	0.1422	0.041*	
C8	0.9661 (4)	0.2567 (4)	0.1403 (4)	0.0312 (10)	
C9	1.0165 (4)	0.4106 (5)	0.1642 (4)	0.0329 (10)	
H9A	1.0899	0.4188	0.1373	0.039*	
H9B	0.9650	0.4848	0.1274	0.039*	
C10	1.0470 (4)	0.1384 (4)	0.1955 (4)	0.0351 (11)	
H10A	1.1204	0.1438	0.1685	0.042*	
H10B	1.0145	0.0411	0.1787	0.042*	

### Atomic displacement parameters (Å^2^)

**Table:**

	*U*^11^	*U*^22^	*U*^33^	*U*^12^	*U*^13^	*U*^23^
Br1	0.0643 (4)	0.0367 (3)	0.0492 (3)	−0.0179 (2)	0.0078 (3)	−0.0076 (2)
Br2	0.0501 (3)	0.0462 (3)	0.0589 (4)	0.0088 (2)	0.0049 (3)	0.0190 (3)
Br3	0.0371 (3)	0.0639 (3)	0.0582 (4)	−0.0055 (2)	0.0214 (3)	−0.0025 (3)
Br4	0.0573 (3)	0.0577 (3)	0.0292 (3)	−0.0008 (2)	0.0054 (2)	−0.0053 (2)
C1	0.033 (2)	0.026 (2)	0.035 (3)	−0.0085 (18)	0.005 (2)	−0.004 (2)
C2	0.031 (2)	0.046 (3)	0.032 (3)	−0.006 (2)	0.002 (2)	−0.001 (2)
C3	0.030 (2)	0.028 (2)	0.034 (3)	0.0008 (18)	0.002 (2)	0.007 (2)
C4	0.043 (3)	0.039 (3)	0.029 (3)	−0.007 (2)	0.009 (2)	0.003 (2)
C5	0.030 (2)	0.036 (2)	0.036 (3)	−0.0030 (19)	0.012 (2)	−0.002 (2)
C6	0.042 (3)	0.032 (2)	0.032 (3)	0.000 (2)	0.005 (2)	−0.007 (2)
C7	0.029 (2)	0.037 (2)	0.036 (3)	−0.0058 (19)	0.006 (2)	−0.001 (2)
C8	0.038 (2)	0.034 (2)	0.020 (2)	−0.0029 (19)	−0.001 (2)	−0.0001 (19)
C9	0.036 (2)	0.034 (2)	0.028 (2)	−0.0040 (19)	0.005 (2)	0.003 (2)
C10	0.037 (2)	0.031 (2)	0.039 (3)	−0.0036 (19)	0.010 (2)	−0.004 (2)

### Geometric parameters (Å, °)

**Table:**

Br1—C1	1.967 (4)	C4—H4B	0.9700
Br2—C3	1.969 (4)	C5—C6	1.526 (6)
Br3—C5	1.974 (4)	C5—C7	1.532 (6)
Br4—C8	1.964 (4)	C6—H6A	0.9700
C1—C6	1.517 (5)	C6—H6B	0.9700
C1—C9	1.520 (6)	C7—C8	1.525 (6)
C1—C2	1.529 (6)	C7—H7A	0.9700
C2—C3	1.522 (6)	C7—H7B	0.9700
C2—H2A	0.9700	C8—C10	1.522 (6)
C2—H2B	0.9700	C8—C9	1.527 (6)
C3—C10	1.522 (6)	C9—H9A	0.9700
C3—C4	1.537 (6)	C9—H9B	0.9700
C4—C5	1.522 (6)	C10—H10A	0.9700
C4—H4A	0.9700	C10—H10B	0.9700
			
C6—C1—C9	110.7 (4)	C1—C6—C5	107.4 (3)
C6—C1—C2	110.4 (4)	C1—C6—H6A	110.2
C9—C1—C2	110.6 (3)	C5—C6—H6A	110.2
C6—C1—Br1	107.6 (3)	C1—C6—H6B	110.2
C9—C1—Br1	109.0 (3)	C5—C6—H6B	110.2
C2—C1—Br1	108.4 (3)	H6A—C6—H6B	108.5
C3—C2—C1	107.3 (3)	C8—C7—C5	107.0 (3)
C3—C2—H2A	110.3	C8—C7—H7A	110.3
C1—C2—H2A	110.3	C5—C7—H7A	110.3
C3—C2—H2B	110.3	C8—C7—H7B	110.3
C1—C2—H2B	110.3	C5—C7—H7B	110.3
H2A—C2—H2B	108.5	H7A—C7—H7B	108.6
C2—C3—C10	110.9 (4)	C10—C8—C7	110.7 (3)
C2—C3—C4	110.1 (3)	C10—C8—C9	111.0 (3)
C10—C3—C4	110.6 (4)	C7—C8—C9	110.2 (3)
C2—C3—Br2	108.7 (3)	C10—C8—Br4	108.4 (3)
C10—C3—Br2	108.9 (3)	C7—C8—Br4	108.1 (3)
C4—C3—Br2	107.4 (3)	C9—C8—Br4	108.3 (3)
C5—C4—C3	106.9 (3)	C1—C9—C8	107.2 (3)
C5—C4—H4A	110.3	C1—C9—H9A	110.3
C3—C4—H4A	110.3	C8—C9—H9A	110.3
C5—C4—H4B	110.3	C1—C9—H9B	110.3
C3—C4—H4B	110.3	C8—C9—H9B	110.3
H4A—C4—H4B	108.6	H9A—C9—H9B	108.5
C4—C5—C6	110.5 (4)	C8—C10—C3	107.3 (3)
C4—C5—C7	111.1 (3)	C8—C10—H10A	110.3
C6—C5—C7	110.3 (4)	C3—C10—H10A	110.3
C4—C5—Br3	108.8 (3)	C8—C10—H10B	110.3
C6—C5—Br3	107.3 (3)	C3—C10—H10B	110.3
C7—C5—Br3	108.9 (3)	H10A—C10—H10B	108.5
			
C6—C1—C2—C3	61.7 (5)	C4—C5—C7—C8	−61.2 (4)
C9—C1—C2—C3	−61.2 (4)	C6—C5—C7—C8	61.6 (4)
Br1—C1—C2—C3	179.3 (3)	Br3—C5—C7—C8	179.0 (3)
C1—C2—C3—C10	61.1 (4)	C5—C7—C8—C10	61.3 (4)
C1—C2—C3—C4	−61.7 (5)	C5—C7—C8—C9	−61.9 (4)
C1—C2—C3—Br2	−179.2 (3)	C5—C7—C8—Br4	179.8 (3)
C2—C3—C4—C5	61.8 (5)	C6—C1—C9—C8	−61.7 (4)
C10—C3—C4—C5	−61.2 (4)	C2—C1—C9—C8	61.1 (4)
Br2—C3—C4—C5	−179.9 (3)	Br1—C1—C9—C8	−179.9 (3)
C3—C4—C5—C6	−61.7 (4)	C10—C8—C9—C1	−61.1 (4)
C3—C4—C5—C7	61.0 (4)	C7—C8—C9—C1	61.9 (4)
C3—C4—C5—Br3	−179.2 (3)	Br4—C8—C9—C1	180.0 (3)
C9—C1—C6—C5	61.4 (5)	C7—C8—C10—C3	−62.0 (4)
C2—C1—C6—C5	−61.4 (5)	C9—C8—C10—C3	60.8 (4)
Br1—C1—C6—C5	−179.6 (3)	Br4—C8—C10—C3	179.7 (3)
C4—C5—C6—C1	61.8 (5)	C2—C3—C10—C8	−60.8 (4)
C7—C5—C6—C1	−61.3 (4)	C4—C3—C10—C8	61.7 (4)
Br3—C5—C6—C1	−179.7 (3)	Br2—C3—C10—C8	179.5 (3)
